# The relevance of multiple clinical specimens in the diagnosis of monkeypox virus, Spain, June 2022

**DOI:** 10.2807/1560-7917.ES.2022.27.33.2200598

**Published:** 2022-08-18

**Authors:** Cristina Veintimilla, Pilar Catalán, Roberto Alonso, Darío García de Viedma, Laura Pérez-Lago, María Palomo, Alejandro Cobos, Teresa Aldamiz-Echevarria, Patricia Muñoz

**Affiliations:** 1 Clinical Microbiology and Infectious Diseases, Hospital General Universitario Gregorio Marañón, Madrid, Spain; 2 Instituto de Investigación Sanitaria Hospital Gregorio Marañón, Madrid, Spain; 3 CIBER Enfermedades Respiratorias- CIBERES (CB06/06/0058), Madrid, Spain; 4 Medicine Department, School of Medicine, Universidad Complutense de Madrid, Madrid, Spain; 5 Centro de Investigación Biomédica en Red de Enfermedades Infecciosas (CIBERINFEC), Instituto de Salud Carlos III, Madrid, Spain

**Keywords:** Monkeypox virus, real-time PCR diagnosis

## Abstract

A monkeypox virus (MPXV) outbreak has been ongoing worldwide since May 2022. The role of specimens other than skin lesions for MPXV diagnosis is unknown. We evaluated 140 different clinical specimens by real-time PCR. The highest positivity rates (97%) were from skin lesions of any part of the body, followed by plasma, pharyngeal and anal swabs. Testing specimens from multiple sites may improve the sensitivity and reduce false-negative test results.

Current evidence suggests that monkeypox virus (MPXV) is acquired by inoculation of the virus into mucosal surfaces or skin through close physical contact. Most cases have been reported among men who have sex with men, raising the possibility of a rapid increase of cases in the context of close physical contact during sexual activity [1,2].

Skin swabs from vesicles, ulcers or crusted lesions are the standard specimens used for the detection of MPXV. However, there is a lack of information about the role that other different specimens may have in MPXV diagnosis [1].To guide sampling strategies, we evaluated the distribution of MPXV among different clinical specimens of patients with suspected MPXV infection.

## Patient samples

We obtained demographical and clinical data from electronic medical records and prospective clinical questionnaires in a cohort of patients with clinical suspicion of MPXV from 27 May to 24 June 2022 in Madrid, Spain. We collected systematically samples from skin lesions and plasma specimens. Anal and oropharyngeal swabs were also collected, depending on the reported type of sexual intercourse or the patient’s symptoms (sore throat or anal pain).

Continuous variables were described using median, mean and interquartile range (IQR) and categorical variables as frequencies and percentages. Association between categorical variables was evaluated by chi-squared test. Stata IC 15.0 (College Station, StataCorp, United States) software was used for statistical assessment.

**Figure 1:**
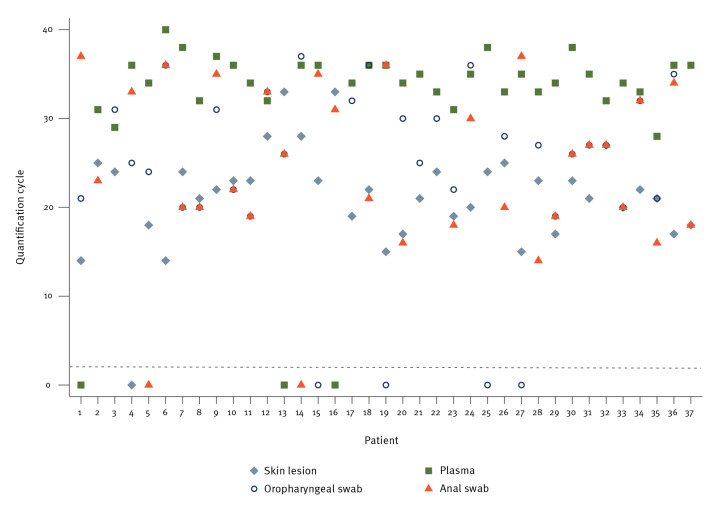
Distribution of diagnostic specimens among 37 patients and Cq values for monkeypox virus PCR, Madrid, Spain, 27 May–24 June 2022 (n = 140 samples)

During the study period, 140 clinical specimens were collected from 37 patients (3–4 specimens per patient) as well as their corresponding quantification cycle (Cq) values (Figure 1). The included patients were men who have sex with men, with a median age of 31 years (IQR: 29–43). The collected specimens were 37 samples from skin lesions, 37 plasma samples, 34 oropharyngeal swabs and 32 anal swabs. According to symptoms reported at the time of sample collection, seven patients had proctitis and anal lesions (Cq range in anal swabs: 18─33) and four patients had sore throat and oropharyngeal lesions (Cq range in oropharyngeal swabs: 21─30) (Table 1).

The specimens under the dashed line were negative for monkeypox virus.

**Table 1:** Characteristics of monkeypox patients, Madrid, Spain, 27 May–24 June (n = 37)

Patients	Days from symptom onset to sampling	Skin lesions Cq values	Plasma Cq values	Oropharyngeal swabs Cq values	Anal swabs Cq value
Patients with sore throat					
21	2	21	33	25	N/A
22	4	24	36	30	N/A
30	3	23	40	26	26
35	3	21	32	21	16
Patients with Proctitis and anal lesions					
2	5	25	Negative	N/A	23
4	5	Negative	31	25	33
8	7	21	34	20	20
10	1	23	34	22	22
13	4	33	35	26	26
29	6	17	32	19	19
37	10	18	33	N/A	18
Patients without sore throat or proctitis/anal lesions					
1	N/A	14	Negative	21	37
3	3	24	34	31	N/A
5	5	18	29	24	Negative
6	2	14	36	36	36
7	4	24	36	20	20
9	2	22	36	31	35
11	8	23	36	19	19
12	4	28	36	33	33
14	4	28	33	37	Negative
15	3	23	35	Negative	35
16	3	33	36	N/A	31
17	6	19	31	32	N/A
18	3	22	38	36	21
19	2	15	35	Negative	36
20	3	17	33	30	16
23	5	19	28	22	18
24	5	20	36	36	30
25	15	24	34	Negative	N/A
26	3	25	32	28	20
27	7	15	Negative	Negative	37
28	2	23	37	27	14
31	8	21	38	27	27
32	3	27	34	27	27
33	4	20	38	20	20
34	2	22	35	32	32
36	2	17	34	35	34

N/A: not available.

## PCR analysis

Samples were collected and carried in viral transport medium to preserve them. All specimens from the same patient were tested simultaneously just after being obtained. Samples were inactivated with 4:1 volume of NUCLISENS easyMAG Lysis Buffer reagent before nucleic acid extraction with an EMAG instrument (BioMérieux). For molecular analyses, we used a real-time PCR, LightMix Modular Monkeypox Virus Kit (TIB MOLBIOL-Roche) for specific detection of MPXV (a 106 bp long fragment from the J2L/J2R gene from the virus is amplified with specific primers and detected with a HEX-labelled hydrolysis probe). Cut-off Cq ≤ 40 were considered as positive, Cq ≥ 41–45 as indeterminate and Cq > 45 or absence of amplification as negative. Reactions were carried out in a CFX96 Touch Real-Time PCR Detection System thermal cycler (BIO-RAD).

Of the 140 collected samples, 10 were negative for MPXV (four oropharyngeal, three plasma, two anal and one skin lesion sample). The range of days between symptom onset and sampling was: 1–15 days. One patient (#4) had a negative MPXV result in skin lesions but was positive in oropharyngeal, anal and plasma samples with Cq values of 25, 33 and 36, respectively, on day 5 after symptom onset. Seven of the 34 oropharyngeal swabs were taken from patients presenting vesicular lesions (five pharyngeal, one tongue and one palate), and 10 of 32 anal swabs were from patients with anal lesions.

**Figure 2:**
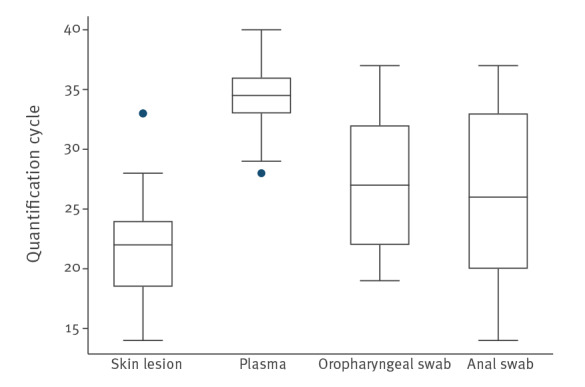
Quantification cycle distribution boxplot, monkeypox virus PCR from different specimens, Madrid, Spain, 27 May–24 June 2022 (n = 130 samples)

We found the highest positivity rate (97%) and viral loads (Cq range: 14–33) in samples taken from skin lesions of any part of the body. In contrast, plasma specimens achieved a high positivity rate (91.9%) with the lowest viral load (Cq range: 28–40) (Figure 2). We explored the correlation between days of symptoms and plasma Cq values without statistical significance (p = 0.834) In the two patients with the longest time from symptom onset (10 and 15 days), Cq values were not higher (Table 1).

In the box and whisker plots, the boxes contain 50% of the sample data, with the median indicated by a horizontal line. The whiskers represent the minimum and maximum values. Blue dots represent outliners values.

Data points for negative results are not included in the plot (n = 1 skin lesion swabs, n = 3 plasma samples, n = 4 oropharyngeal swabs and n = 2 anal swabs). None of our samples had an indeterminate result.

The positivity rates for the remaining specimens were 88% in oropharyngeal swabs (Cq range: 19–37) and 94% in anal swabs (Cq range: 14–37) (Table 2).

**Table 2:** Positivity rates obtained by performing real-time monkeypox PCR, Madrid, Spain, 27 May–24 June (n = 140 samples)

Specimens and values	Skin lesion^a^ (n = 37)		Plasma (n = 37)		Oropharyngeal swab (n = 34)		Anal swab (n = 32)	
	n	%	n	%	n	%	n	%
Positive PCR results	36	97	34	91.9	30	88	30	93.8
Mean Cq (SD)	21.8 (4.6)		34.4 (2.6)		27.3 (5.7)		26 (7.4)	
Cq range	14–33		28–40		19–37		14–37	
95% CI	20.2–23.3		33.5–35.3		25.1–29.4		23.3–28.8	

CI: confidence interval; Cq: quantification cycle**;** SD: standard deviation.

^a^ Includes: vesicula, pustular or crusted lesions.

## Discussion

Monkeypox virus is a zoonotic pathogen of the *Orthopoxvirus* genus and *Poxviridae* family. The current multinational outbreak in areas not endemic for monkeypox was first reported in May 2022 [3]. On 19 May, Portugal reported the first genome sequence of the MPXV associated with the ongoing outbreak and confirmed that it belongs to the West African clade [4]. In our laboratory, the MPXV genome sequences from the first two patients were assigned to the same clade [5].

In this study, we evaluated 140 different clinical specimens from 37 patients with suspected MPXV infection. Skin lesions showed the highest positivity rate and viral load. Interestingly, the majority of our plasma samples tested positive for MPXV, indicating that the patients were viraemic at the time of samples collection. The observed Cq values indicate low viral load, suggesting that these patients may have been positive before the appearance of skin lesions. Therefore, we could propose that performing the detection of MPXV in plasma specimens from close contacts without skin lesions might allow early diagnosis and control of the transmission chain; nevertheless this should be further investigated.

We found no statistically significant association between time since symptom onset and plasma Cq values. This could be due to the subjective identification of the beginning on systemic symptoms referred by the patients and to the time point of testing. In a previous report, blood samples were tested in two patients on different days and also showed high Cq values [6]. To our knowledge, there is a lack of evidence about the detection of MPXV DNA in blood samples and its epidemiological and clinical significance [7,8].

The oropharyngeal and anal swabs were also frequently positive for MPXV with Cq, similar to those in skin lesions. Although some of our patients had vesicular lesions in oropharyngeal and anal areas, most positive samples did not come from patients with visible lesions in those areas at the time of testing (27/34 oropharyngeal and 22/32 anal samples). Therefore, these additional specimens could be relevant, depending on the symptoms and the potential risks during the close physical contact (oral or anal intercourse), in reaching an early MPXV diagnosis in patients without skin lesions. Recent reports demonstrated positive MPXV PCR detection with Cq values < 30 in some other sample types such as seminal fluid, faeces and nasopharynx which could be involved in transmission of the disease [7]. Other authors also describe nasopharyngeal swab and blood specimens as useful for diagnosis. They further indicate that anal or rectal swabs should be considered for individuals presenting with anal pain or proctitis [9]. In our study, patient (#4) who did not present skin lesions was diagnosed from additional specimens, hence taking several samples may be important in cases with exposure history.

We are aware of some limitations of this study such as the inability to confirm positive PCR results and their duration with viral culture assays to demonstrate ongoing viable viral shedding. Also, to address the best utility of blood samples, prospective studies at different time points of the disease and before the development of lesions are needed.

## Conclusion

Our results contribute to understanding MPXV transmission and the importance of testing specimens from multiple sites in situations with relevant exposure history and e.g. missing skin lesions, which may improve diagnostic sensitivity and reduce false-negative test results.
